# Effect of mindfulness on anxiety and depression in insomnia patients: A systematic review and meta-analysis

**DOI:** 10.3389/fpsyt.2023.1124344

**Published:** 2023-03-02

**Authors:** Hangyu Li, Wanli Qin, Nannan Li, Shixing Feng, Junqi Wang, Yuan Zhang, Tianyi Wang, Chenlu Wang, Xuanyi Cai, Wen Sun, Yang Song, Dongran Han, Yixing Liu

**Affiliations:** ^1^School of Life and Science, Beijing University of Chinese Medicine, Beijing, China; ^2^Xiyuan Hospital, China Academy of Chinese Medical Sciences, Beijing, China; ^3^Department of Neurology, Dongfang Hospital Beijing University of Chinese Medicine, Beijing, China; ^4^Dongzhimen Hospital Beijing University of Chinese Medicine, Beijing, China; ^5^School of Management, Beijing University of Chinese Medicine, Beijing, China; ^6^School of Humanities, Beijing University of Chinese Medicine, Beijing, China

**Keywords:** mindfulness, insomnia, anxiety, depression, meta-analysis

## Abstract

**Background:**

As a common clinical symptom, insomnia has a high incidence of combined mental illness and it is also a risk factor for the development of depression, anxiety and suicide. As a new concept in the field of health in recent years, mindfulness therapy can improve insomnia, anxiety and depression, which is a new way to solve such diseases.

**Objective:**

This study aims to systematically evaluate the effects of mindfulness compared with conventional treatment on scores of the Hamilton Depression Scale (HAMD), Hamilton Anxiety Scale (HAMA), Self-Rating Depression Scale (SDS) and Self-Rating Anxiety Scale (SAS) in people with insomnia and anxiety-depressive symptoms.

**Methods:**

Articles published before October 2022 were searched from seven databases and included in randomized controlled trials (RCTs) to evaluate mindfulness therapy. The assessment tool of Cochrane bias risk was used to evaluate the methodological quality of the literature. The main outcome indicators were HAMD and HAMA scores, and the secondary outcome indicators were SDS and SAS scores.

**Results:**

Ten randomized controlled trials including 1,058 subjects were systematically evaluated and meta-analyzed in this study. In the main outcome indicators, there was a significant difference between mindfulness therapy and conventional treatment in reducing HAMD score (MD: −3.67, 95% CI: −5.22–2.11, *p* < 0.01) and HAMA score (MD: −3.23, 95% CI: −3.90–2.57, *p* < 0.01). In the secondary outcome indicators, mindfulness therapy also showed a significant difference in reducing SDS scores (MD: −6.49, 95% CI: −6.86–6.11, *p* < 0.01) and SAS scores (MD: −7.97, 95% CI: −9.68–6.27, *p* < 0.01) compared with conventional treatment.

**Conclusion:**

For the people with insomnia, anxiety and depression, the use of conventional treatment with the addition of 4–12 weeks of mindfulness treatment can significantly improve anxiety and depression symptoms of patients. This is a new diagnosis and treatment idea recommended for insomniacs with or without anxiety and depression symptoms. Due to the methodological defects in the included study and the limited sample size of this paper, more well-designed randomized controlled trials are needed for verification.

## 1. Introduction

As a common clinical symptom, insomnia is characterized by difficulty entering or maintaining sleep, and is accompanied by symptoms such as tiredness, fatigue, and dysphoria when awake. The most common comorbidity associated with insomnia is psychiatric illness (1), and the prevalence of insomnia has been reported to be higher in older adults and individuals with mental disorders or chronic illnesses (2). Therefore, insomnia often increases the risk of neuropsychiatric diseases such as anxiety and depression (3), and is a risk factor for the development of depression, anxiety disorders, and suicide. Depression, anxiety, and other bad emotions can aggravate the severity of insomnia. They have a close connection of mutual influence, mutual cause and effect with a high comorbidity rate. Such studies suggest that preemptive therapy for insomnia may reduce the risk of depression (4). In the general population, the frequency of combined insomnia and anxiety disorders was found to be 32.5 percent, while it was 17.3 percent for insomnia with severe depressive disorder (5).

Current pharmacotherapy for insomnia has limitations that may lead to drug dependence and tolerance, and side effects typically include residual daytime sleepiness, acute memory impairment, and ataxia. The use of benzodiazepines has been reported to increase the risk of cognitive impairment and dementia by 50 percent (6). In addition, the potential association between sedative-hypnotic drugs for insomnia and increased mortality is highly controversial. Most investigators agree that changes of monoamine neurotransmitter levels (NE, GABA, 5-HT, DA, etc.) are associated with sleep disturbance and depression (7). Studies have also shown that under strong emotional stress, disorders of the HPA axis and neuro-secretion-immune-metabolic system can lead to insomnia, anxiety and depression (8). Therefore, the Western medicine mostly use the combination of sedation, anti-anxiety and antidepressants for its treatment. But there are many adverse reactions in long-term use with obvious drug resistance. The effect is not significant.

Originating from the East, mindfulness is one of the core Zen methods of Buddhism. It gradually faded the religious implication after the rise of the West and became an important psychotherapy. Mindfulness meditation is a process of openly and consciously engaging in the experience of the present moment. Mindfulness therapy, a new concept in the field of health that has emerged in recent years, has been widely reported in scientific reports and the media, describing the potential benefits of mindfulness on a wide range of topics, from physical and mental health to cognitive, emotional, and interpersonal outcomes (9, 10). One definition of mindfulness is the process that people openly and consciously focus on the experience in the present moment. This conscious process of experiencing the present moment contrasts with much of our daily life, in which we often find ourselves in unconscious thoughts wandering (11) or uncontrolled automatic wandering (12). By comparison, the ability of mindfulness or meditation can be positively correlated with increased happiness in daily life (13). Later, Coelho (14) believed that mindfulness is not only a continuous and conscious deep cognition of the mind (mindfulness awareness), but also a conscious focus on the present experience (mindfulness practice) in an open, caring and clear way.

In recent years, there has been evidence (15, 16) that mindfulness has a significant effect on insomnia, and can also improve symptoms of anxiety and depression caused by insomnia, but evidence-based medicine evidence is still lacking. Since insomnia is often accompanied by a certain degree of anxiety and depression, this study focuses on people with insomnia disorders or poor sleep quality in clinical practice, and in line with the coexistence of anxiety and depression. With the intervention of mindfulness therapy, whether the improvement of anxiety and depression of these people is different from that of conventional treatment.

## 2. Materials and method

This systematic review and meta-analysis followed the PRISMA statement. The review protocol was registered at PROSPERO (Registration number: CRD42022369832).

### 2.1. Search strategies

Computer searches of Embase, PubMed, The Cochrane Library, VIP, WanFang Data, CBM, and CNKI databases were conducted to collect randomized controlled trials related to mindfulness for the treatment of insomnia with anxiety and depression, with a search time frame from database creation to October 2022. The search was conducted using a combination of subject terms and free terms, and was adjusted to the characteristics of each database. References included in the study were also searched to supplement relevant information. Search terms include: insomnia, sleep disorder, anxiety, depression, negative emotion, Mindfulness, and Randomized Controlled Trial etc.

### 2.2. Eligibility criteria

Two authors (JW and WQ) independently screened the eligible clinical trials based on the inclusion criteria as follows:

1. RCTs investigating the mindfulness therapy in the treatment of anxiety and depression in insomnia patients were included.
2. The diagnosis was based on ‘The International Statistical Classification of Diseases and Related Health Problems 10th Revision (ICD-10) (17)’ or ‘Chinese classification of mental disorders Version 3 (CCMD-3) (18)’.
3. Duration of the treatment was more than 4 weeks.
4. The total sample size were more than 30.
5. The primary outcome of the study was the Hamilton Depression Rating Scale (HAMD) and Hamilton Anxiety Rating Scale (HAMA) scores; the secondary outcome was the Self-Rating Depression Scale (SDS) and the Self-Rating Anxiety Scale (SAS).

Randomized controlled trials should be excluded, which did not comply with the above criteria: (a) formulation and dosage of intervention in the treatment, control or united groups were not provided in detail; (b) data incomplete or duplication; (c) another TCM therapy was used.

According to the inclusion and exclusion criteria, two authors (TW and CW) independently searched the databases to obtain the eligible clinical trials. One author (TW) read the full text of the eligible collected articles. Another author (CW) checked the accuracy and completeness of the collected articles. During the process of study selection, two authors solved the disagreements through discussion. If consensus was not reached, a third author (YZ) would step in to discuss resolved differences.

### 2.3. Data items

The following items in the included RCTs were collected: (1) first author; (2) publication year; (3) sample size of patients; (4) specific mindfulness treatment methods used in the intervention group, including treatment frequency and duration; (5) conventional treatment methods used in the control group, including drug therapy, psychological therapy and healthy lifestyle; (6) outcomes on depression and anxiety measures; (7) risk of bias and summaries of major findings. Data for the meta-analysis were extracted separately and included the type of treatment group, mean and SD of pre-and-post-treatment depression and anxiety indicators, and sample size for each outcome.

### 2.4. Types of outcomes measurements

The primary outcome measure of this study was the HAMD and HAMA scales, which were used to assess symptoms of depression and anxiety, respectively. They were independently scored by two trained assessors using conversation and observation, on a scale of 0 (none) to 0 4 (severe), which can better assess the severity of depression and anxiety symptoms. The higher the score, the more severe the psychological distress, depression and anxiety. The secondary outcome measures were SDS and SAS scales, which are characterized by the ease of use, can visually measure and assess the subjective feelings of depression, anxiety and changes in treatment. They are widely applicable and commonly used as self-assessment tools in psychological counseling or psychiatric clinics.

### 2.5. Quality assessment

According to the RoB assessment tool in the Cochrane Handbook, each included study was evaluated independently by two authors (JW and WQ), including the risk of bias in the following aspects: (1) random sequence generation; (2) allocation concealment; (3) blinding of participants and personnel; (4) blinding of outcome assessment; (5) incomplete outcome data; (6) selective reporting; (7) other bias. Based on the Cochrane Assessment Tool, our judgments for these domains were categorized as “low risk of bias,” “high risk of bias,” or “unclear risk of bias.” Disagreements between two authors in the evaluation are resolved through discussion, and when consensus cannot be reached, a decision is made by a third author (SF).

### 2.6. Statistics analysis

Based on the age and duration to execute subgroup analysis, the R 4.2.1 software, and the Review Manager version 5.3 software (Cochrane Collaboration, Nordic Cochrane Centre, Copenhagen, Denmark) were employed to analyze the collected data in this study.

The R 4.2.1 software was used for the statistical analysis. The dichotomous variable was represented as the pooled risk ratios (RRs) with 95% CI. The continuous variable was represented as the mean difference (MD) with 95% CI. I^2^ statistic and the chi-squared test were applied to evaluate the heterogeneity among the included RCTs. If *I^2^* > 50% or *p* < 0.05, it suggested that a significant statistical heterogeneity was observed, and the random-effect model should be employed to evaluate the outcome measures. Otherwise, the fixed-effect model was adopted. If *p* < 0.05, it suggested that there was a significant statistical difference in this meta-analysis.

Regardless of the clinical heterogeneity of the combined outcomes, subgroup analyses were conducted to obtain more clinically meaningful results. Subgroup analyses focused on the primary and secondary outcome scores effects of mindfulness intervention time periods and different age groups.

The funnel plot created by the R 4.2.1 software was employed to evaluate the potential publication bias. Begg’s test and Egger’s test were also used to detect the potential publication bias of the categorical variable and the continuous variable in this meta-analysis, respectively.

## 3. Results

### 3.1. Study selection

**Figure 1:**
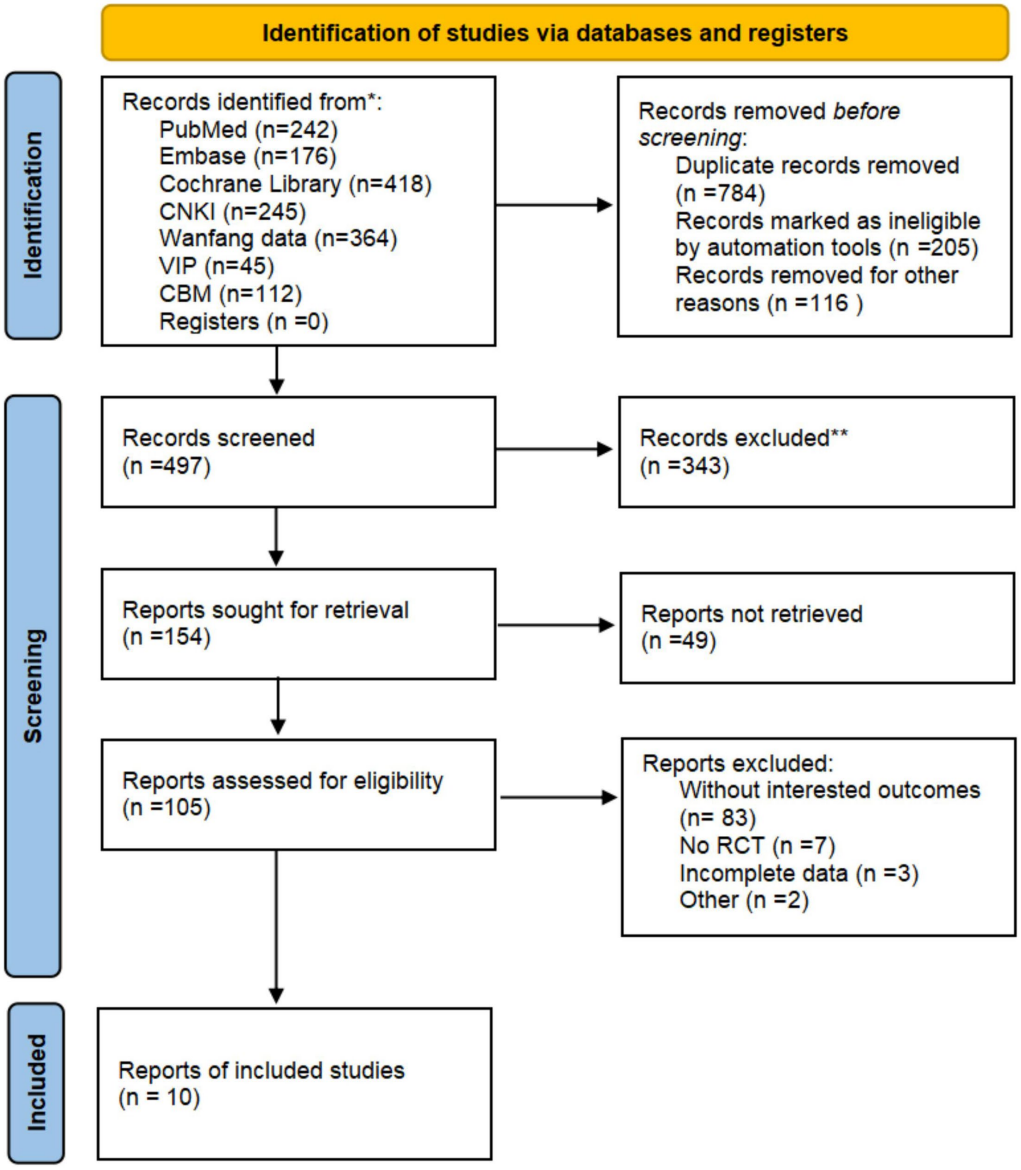
PRISMA flow diagram of the trial selection process.

The process of the study selection for the eligible RCTs is shown in Figure 1. According to the search strategies, 1,602 potential studies were initially identified from the databases. 1,105 studies were excluded because they were not relevant to the present analysis. According to the eligibility criteria, full texts of 105 studies were retrieved for manually screening. Finally, 10 RCTs were included for further quality evaluation and meta-analysis. The detailed process of identification and selection is shown in the PRISMA flow diagram (19) (Figure 1).

### 3.2. Study characteristics

The detailed characteristics of the included 10 RCTs are given in Table 1 (20–29). Ten RCTs, with publication date was from 2017 to 2022, were published in Chinese language journals. The number of insomnia patients with anxiety and depression in the included RCTs ranged from 44 to 160. The duration of the treatment was ranging from 4 to 12 weeks. The interventions used in these articles include mindfulness-based stress reduction therapy, mindfulness yoga relaxation training, mindfulness-based sedentary breathing training, integrative relaxation training combined with exercise therapy, mindfulness meditation training, and mindfulness-based cognitive training. The conventional treatment methods used in the control group included anti-anxiety-depression and tranquilizer medications, psychological therapy and maintaining a healthy lifestyle. In 10 RCTs, HAMD and HAMA were selected as primary outcomes, while SDS and SAS were selected as secondary outcomes.

**Table 1:** The characteristics of included studies in the meta-analysis.

Studies	Sample size	Ages	Intervention Group	Treatment Duration	Control Group	Insomnia Measure	Depression Measure	Anxiety Measure
Wei CY et al. 2017 (20)	T: 80/C:80	T: 57.1 ± 7.9/C: 58.3 ± 8.7	Mindfulness Based Stress Reduction(MBSR)	week intervention; 50-min weekly	Conventional treatment	PSQI	SDS	SAS
Dang QN et al. 2017 (21)	T: 22/C: 22	T: 31.4 ± 6.75/C: 28.3 ± 7.25	Mindfulness Yoga Relaxation Training	week intervention; 90 min per training, 3 times a week	Conventional treatment	PSQI	HAMD	HAMA
Li LX et al. 2018 (22)	T: 40/C: 40	T: 52.6 ± 1.2/C: 52.3 ± 1.1	Mindfulness Based Stress Reduction(MBSR)	6-week intervention; 3 times a day	Conventional treatment	PSQI	SDS	SAS
Zhang HF et al. 2020 (23)	T: 80/C: 80	T: 47.3 ± 8.6/C: 46.7 ± 8.4	Mindfulness Based Stress Reduction(MBSR)	6-week intervention	Conventional treatment	PSQI、ISI	SDS	SAS
Yu YT et al. 2020 (24)	T: 38/C: 38	T: 46.8 ± 3.6/C: 46.3 ± 3.4	Mindfulness Based Stress Reduction(MBSR)	8-week intervention; twice a day, 5 times a week	Conventional treatment	PSQI	SDS	HAMA、SAS
Yang LJ et al. 2020 (25)	T: 68/U: 68	T: 41.02 ± 5.16/C: 42.14 ± 5.32	Mindfulness Based Stress Reduction(MBSR)	8-week intervention; 60 min per training, twice a week	Conventional treatment	PSQI	HAMD	HAMA
Liu L et al. 2021 (26)	T: 80/C: 80	T: 46.66 ± 15.05/C: 49.20 ± 13.38	Mindfulness Meditation Breathing Training	4-week intervention; 1–2 times a day	Conventional treatment	PSQI	HAMD	HAMA
Zhai N et al. 2021 (27)	T: 38 C: 38	T: 37.68 ± 7.59/C: 38.23 ± 7.84	Comprehensive relaxation training combined with exercise therapy	4-week intervention; Comprehensive relaxation training 40 min per training, twice a day; exercise therapy 30 min a day, 5 times a week	Conventional treatment	PSQI	HAMD	HAMA
Zhong YY et al. 2022 (28)	T: 40/C: 40	T: 47.9 ± 6.3 C: 48.5 ± 5.2	Mindfulness Meditation Training	8-week intervention; 40 min per training, twice a week	Conventional treatment	PSQI	SDS	SAS
Zhang QB et al. 2022 (29)	T: 43/C: 43	T: 20.15 ± 0.78/C: 20.11 ± 0.85	Mindfulness Cognitive Training	12-week intervention	Conventional treatment	PSQI	SDS	SAS

### 3.3. Risk of bias in individual studies

**Figure 2:**
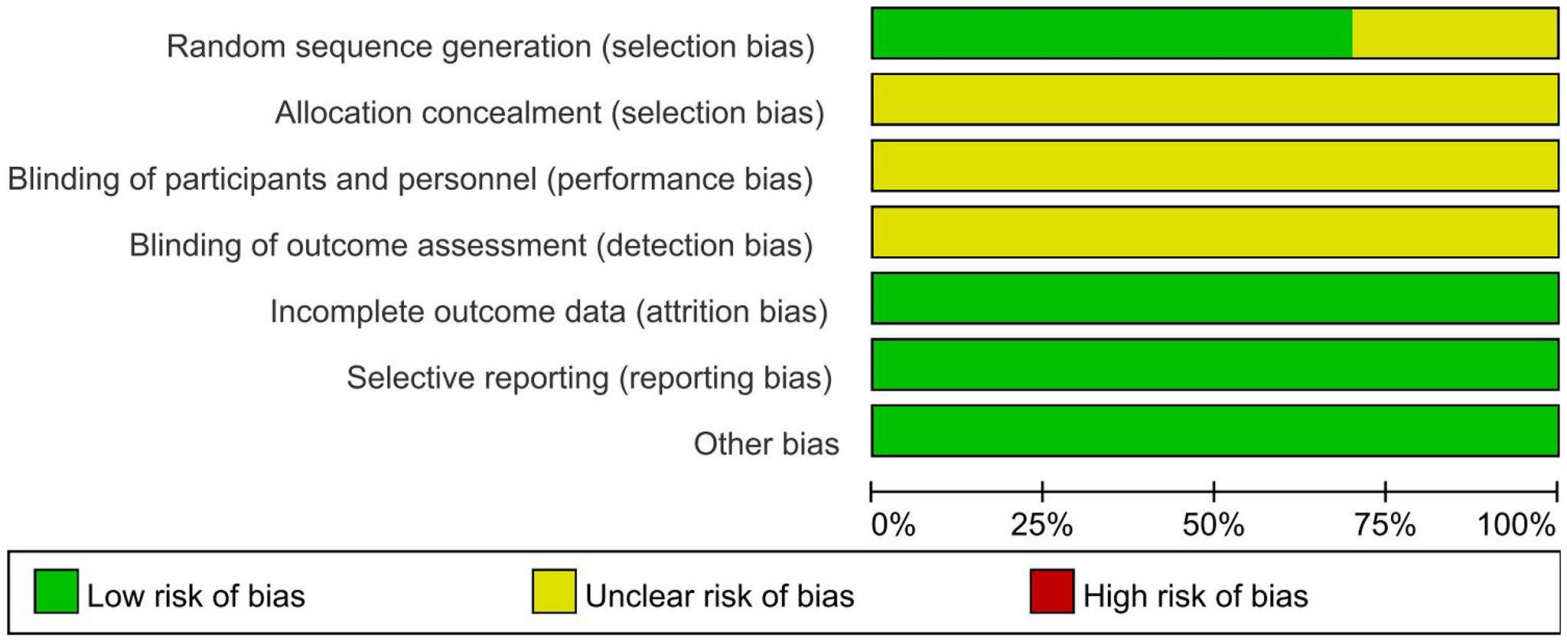
The risk of bias for the included studies.

**Figure 3:**
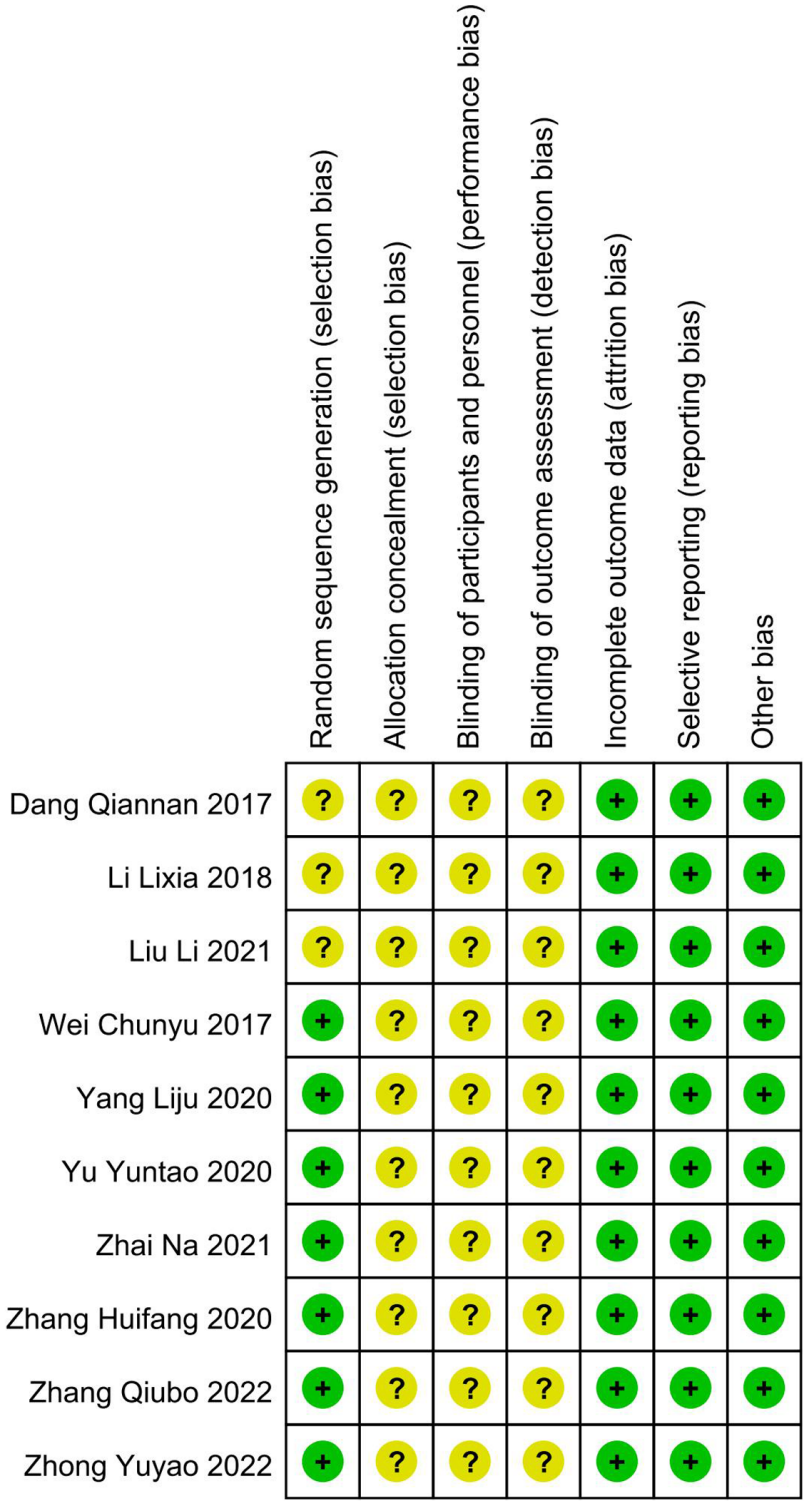
Risk of bias summary: review authors’ judgments about each risk of bias item for each included study.

We summarize the risk of bias assessment for each of the included studies in Figures 2, 3.

#### 3.3.1. Random sequence generation

Seven studies (20, 23–25, 27–29) had a low risk of randomization bias, which were described as RCTs and indicated that randomization grouping was performed. Three studies (21, 22, 26) did not mention the grouping method, which we assessed as “unknown.”

#### 3.3.2. Allocation concealment

None of the 10 studies (20–29) we included mentioned randomized concealment and were all assessed as “unknown.”

#### 3.3.3. Blinding of participants and personnel and outcome assessment

The intervention of this study was mindfulness treatment, and the blinding method of subjects was not mentioned in the study design, so 10 studies (20–29) were assessed as “unknown.” In addition, all trials (20–29) were rated as having an unclear risk of assay bias because no blinding of clinicians, analysts, or data collectors was reported.

#### 3.3.4. Incomplete outcome data

All studies (20–29) were assessed as low risk for outcome reporting.

#### 3.3.5. Selective reporting

Although all studies (20–29) did not have a trial protocol, reported planned outcomes in the study and therefore we judged them to be low risk.

#### 3.3.6. Other potential sources

No other factors affecting bias were mentioned in any of the 10 studies (20–29), so we judged it to be low risk.

### 3.4. Effects of the mindfulness

#### 3.4.1. HAMD

##### 3.4.1.1. Meta-analysis

**Figure 4:**
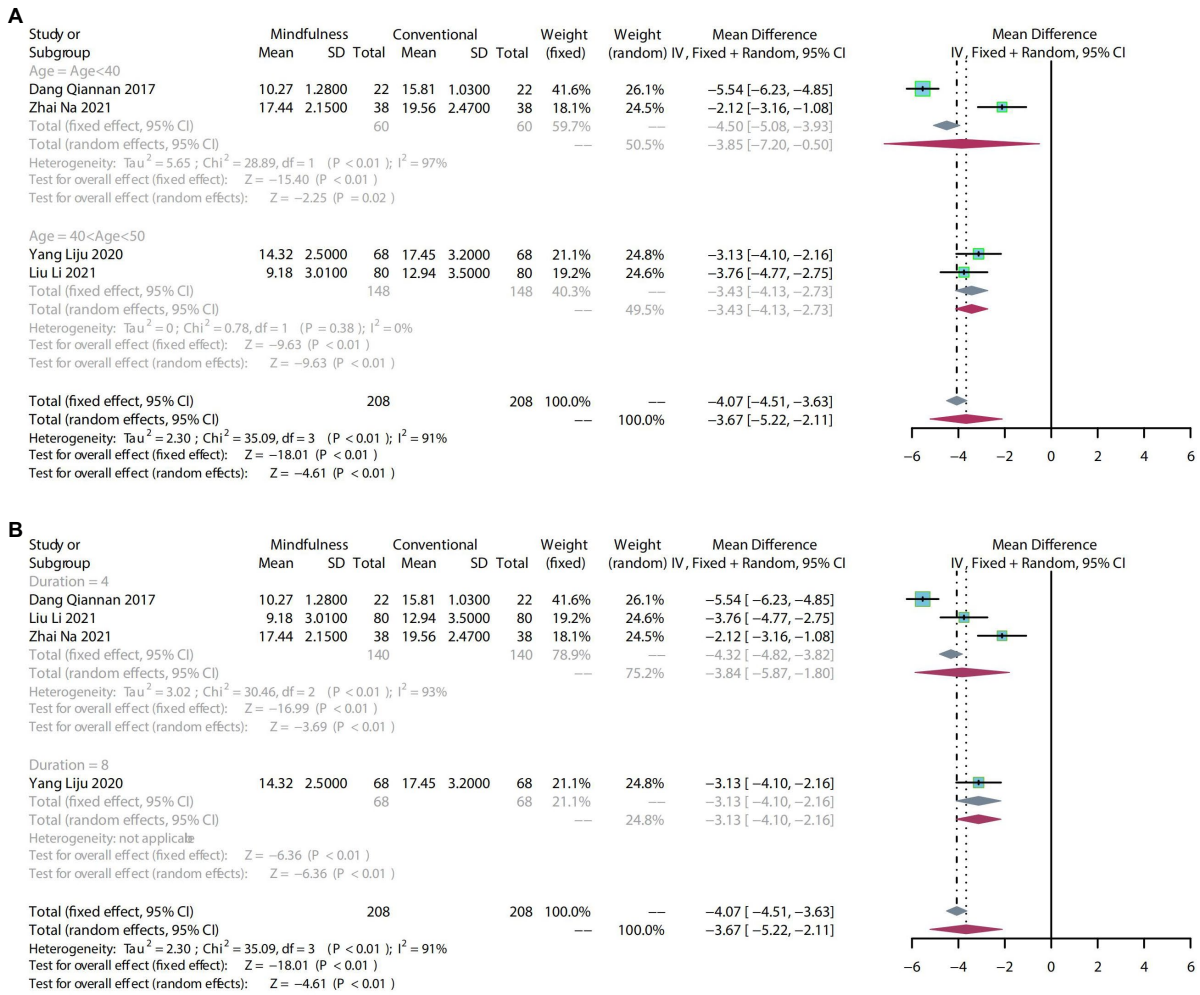
**(A)** forest plot showed the effect of mindfulness versus conventional treatment on HAMD scores in subgroups stratified according to age. **(B)** Forest plot showed the effect of mindfulness versus conventional treatment on HAMD scores in subgroups stratified according to duration of treatment.

To compare the effects of mindfulness therapy for depression in people with insomnia, anxiety and depression, four RCTs including 416 participants were analyzed to assess the endpoint HAMD score. Due to the high heterogeneity (*p* < 0.01, *I^2^* = 91%), a random-effects model was used. Result indicated that a clear difference in reducing HAMD scores between mindfulness treatment and conventional treatment (MD: −3.67, 95% CI: −5.22–−2.11, *p* < 0.01; Figure 4).

##### 3.4.1.2. Subgroup analysis

As the HAMD scores showed a high degree of heterogeneity (*p* < 0.01, *I^2^* = 91%), we performed subgroup analyses from the age of trial participants and the duration of the treatment to identify sources of heterogeneity and provide more valuable evidence for clinical practice. The same subgroup analysis was used in the following outcome indicators.

After subgroup analysis by age, age < 40 (MD: −3.85, 95% CI: −7.20–−0.50, *p* = 0.02 and 40 < age > 50 (MD: −3.43, 95% CI: −4.13–−2.73, *p* < 0.01) demonstrated that mindfulness significantly reduced HAMD scores compared with conventional treatment (Figure 4A).

After subgroup analysis according to the duration of treatment, 4 weeks of mindfulness treatment (MD: −3.84, 95% CI: −5.87–−1.80, *p* < 0.01) was significantly different from conventional treatment. Meanwhile, the impact of 8 weeks of mindfulness treatment (MD: −3.13, 95% CI: −4.10–−2.16, *p* < 0.01) on the HAMD score may require more evidence to support it (Figure 4B).

#### 3.4.2. HAMA

##### 3.4.2.1. Meta-analysis

**Figure 5:**
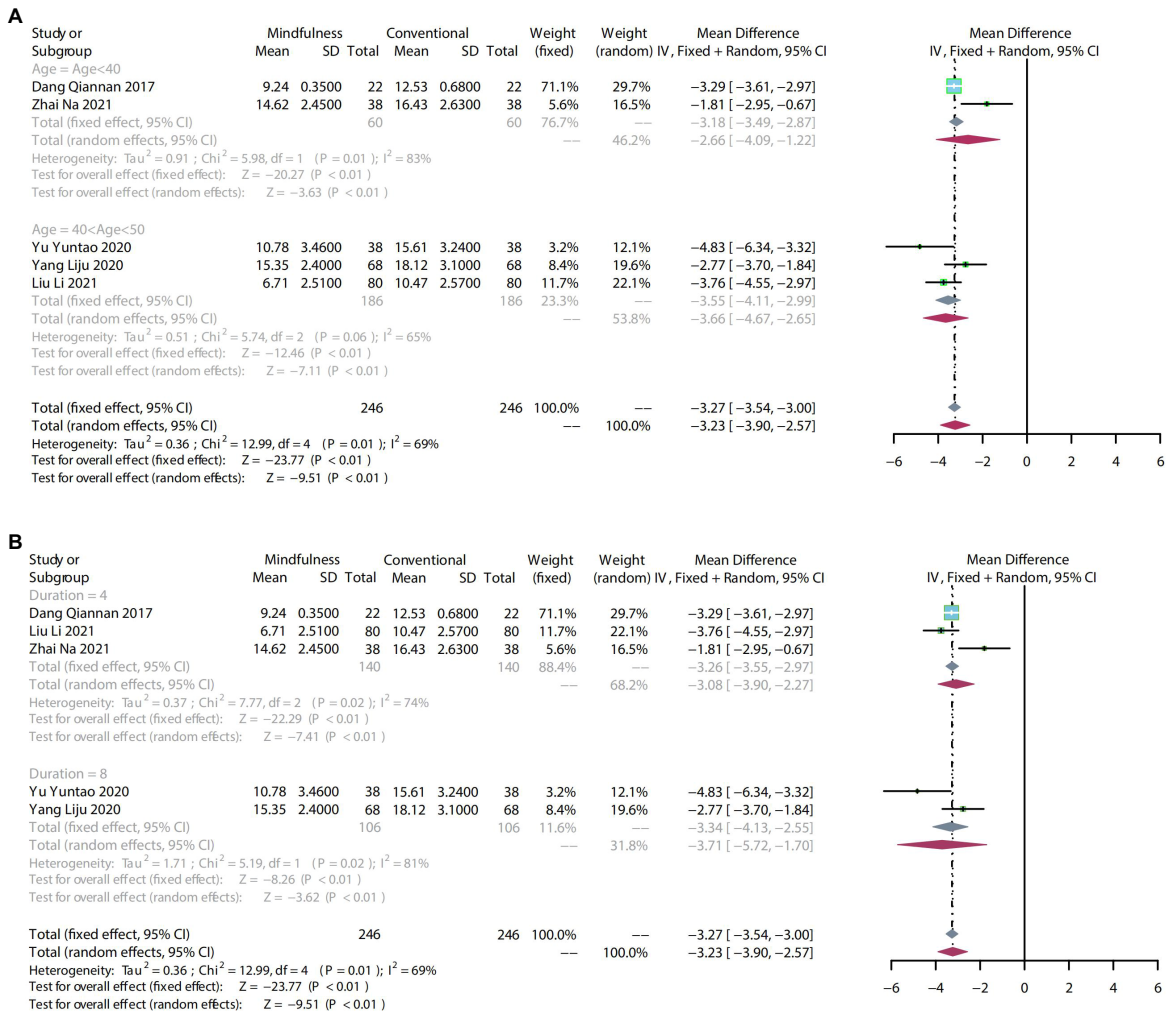
**(A)** forest plot shows the effect of mindfulness versus conventional treatment on HAMA scores in subgroups stratified according to age. **(B)** Forest plot showing the effect of mindfulness versus conventional treatment on HAMA scores in subgroups stratified according to duration of treatment.

To compare the effects of mindfulness therapy for anxiety in people with insomnia, anxiety and depression, five RCTs including 492 participants were analyzed to assess the endpoint HAMA score. Due to moderate heterogeneity (*p* = 0.01, *I^2^* = 69%), a random-effects model was used. The results showed that a clear difference in the reduction of HAMA scores between mindfulness and conventional treatment (MD: −3.23, 95% CI: −3.90–−2.57, *p* < 0.01; Figure 5).

##### 3.4.2.2. Subgroup analysis

The HAMA scores showed high heterogeneity (*p* = 0.01, *I^2^* = 69%).

After subgroup analysis by age, age < 40 (MD: −2.66, 95% CI: −4.09–−1.22, *p* < 0.01) and 40 < age > 50 (MD: −3.66, 95% CI: −4.67–−2.65, *p* < 0.01) demonstrated that mindfulness significantly reduced HAMA scores compared with conventional treatment (Figure 5A).

After subgroup analysis according to duration of treatment, 4-week mindfulness treatment (MD: −3.08, 95% CI: −3.90–−2.27, *p* < 0.01) was significantly different from conventional treatment. Meanwhile, compared with conventional treatment, 8-week mindfulness therapy (MD: −3.71, 95% CI: −5.72–−1.70, *p* < 0.01) on the impact of HAHA scores also varied significantly (Figure 5B).

#### 3.4.3. SDS

##### 3.4.3.1. Meta-analysis

**Figure 6:**
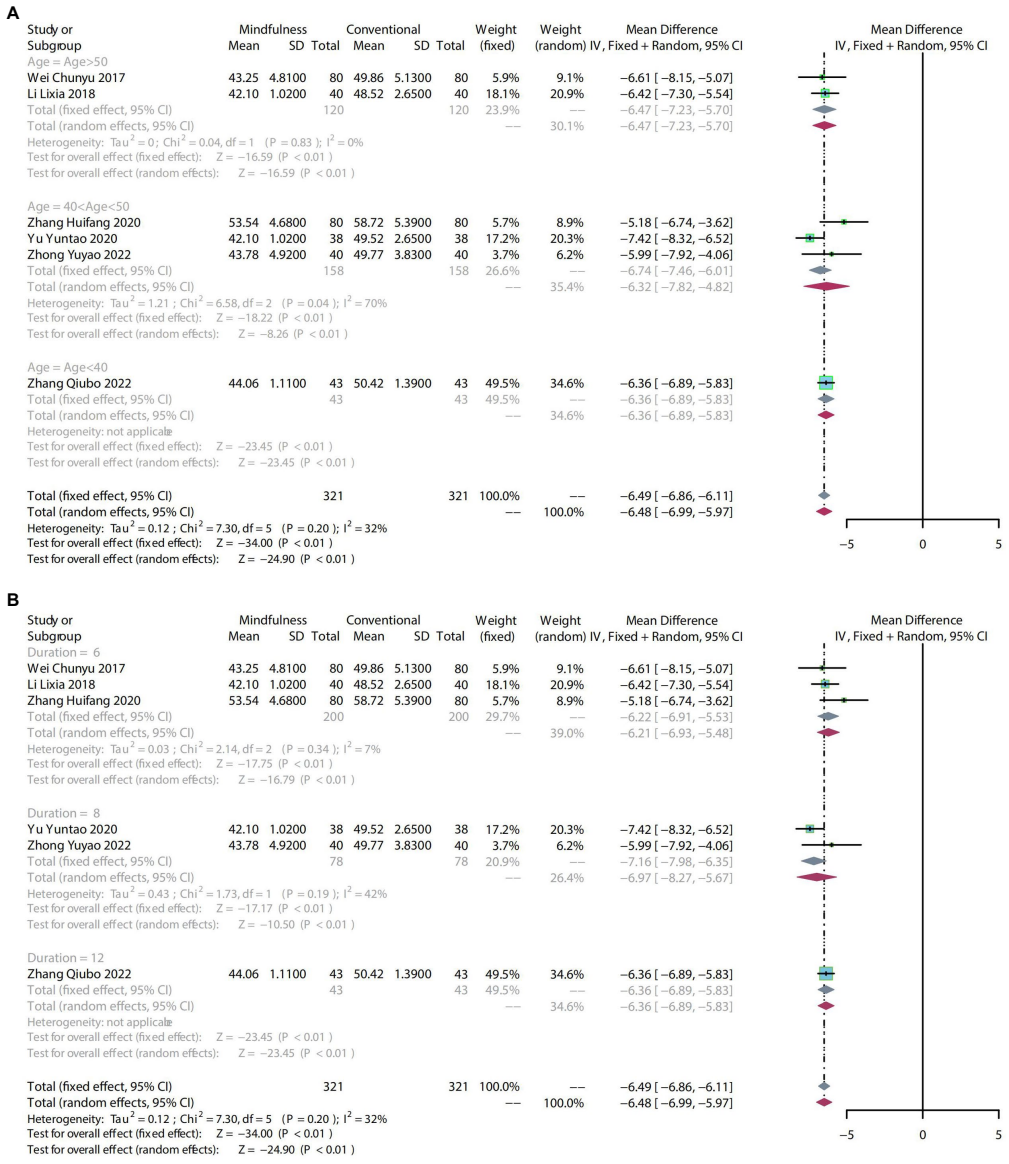
**(A)** forest plot shows the effect of mindfulness versus conventional treatment on SDS scores in subgroups stratified according to age. **(B)** The forest plot shows the effect of mindfulness versus conventional treatment on SDS scores in subgroups stratified according to duration of treatment.

To compare the effects of mindfulness therapy for depression in people with insomnia, anxiety and depression, six RCTs including 642 participants were analyzed to assess endpoint SDS scores. Due to mild heterogeneity (*p* = 0.20, *I^2^* = 32%), a fixed-effect model was used. The comprehensive results showed a clear difference in the reduction of SDS scores between mindfulness treatment and conventional treatment (MD: −6.49, 95% CI: −6.86–−6.11, *p* < 0.01) (Figure 6).

##### 3.4.3.2. Subgroup analysis

The SDS scores showed mild heterogeneity (*p* = 0.20, *I^2^* = 32%).

After subgroup analysis by age, age > 50 (MD: −6.47, 95% CI: −7.23–−5.70, *p* < 0.01) and 40 < age > 50 (MD: −6.32, 95% CI: −7.82–−4.82, *p* < 0.01) demonstrated a significant difference compared to conventional treatment. For mindfulness therapy with age < 40 (MD: −6.36, 95% CI: −6.89–−5.83, *p* < 0.01), the effect on SDS scores may need more evidence to support it (Figure 6A).

The subgroups were divided into 6 weeks, 8 weeks, and 12 weeks according to the duration of treatment. Six weeks (MD: −6.22, 95% CI: −6.91–−5.53, *p* < 0.01) and 8 weeks of mindfulness therapy (MD: −6.97, 95% CI: −8.27–−5.67, *p* < 0.01) had a significant difference compared to conventional treatment. The 12-week mindfulness treatment (MD: −6.36, 95% CI: −6.89–−5.83, *p* < 0.01) on the impact of SDS scores may require more evidence to support it (Figure 6B).

#### 3.4.4. SAS

##### 3.4.4.1. Meta-analysis

**Figure 7:**
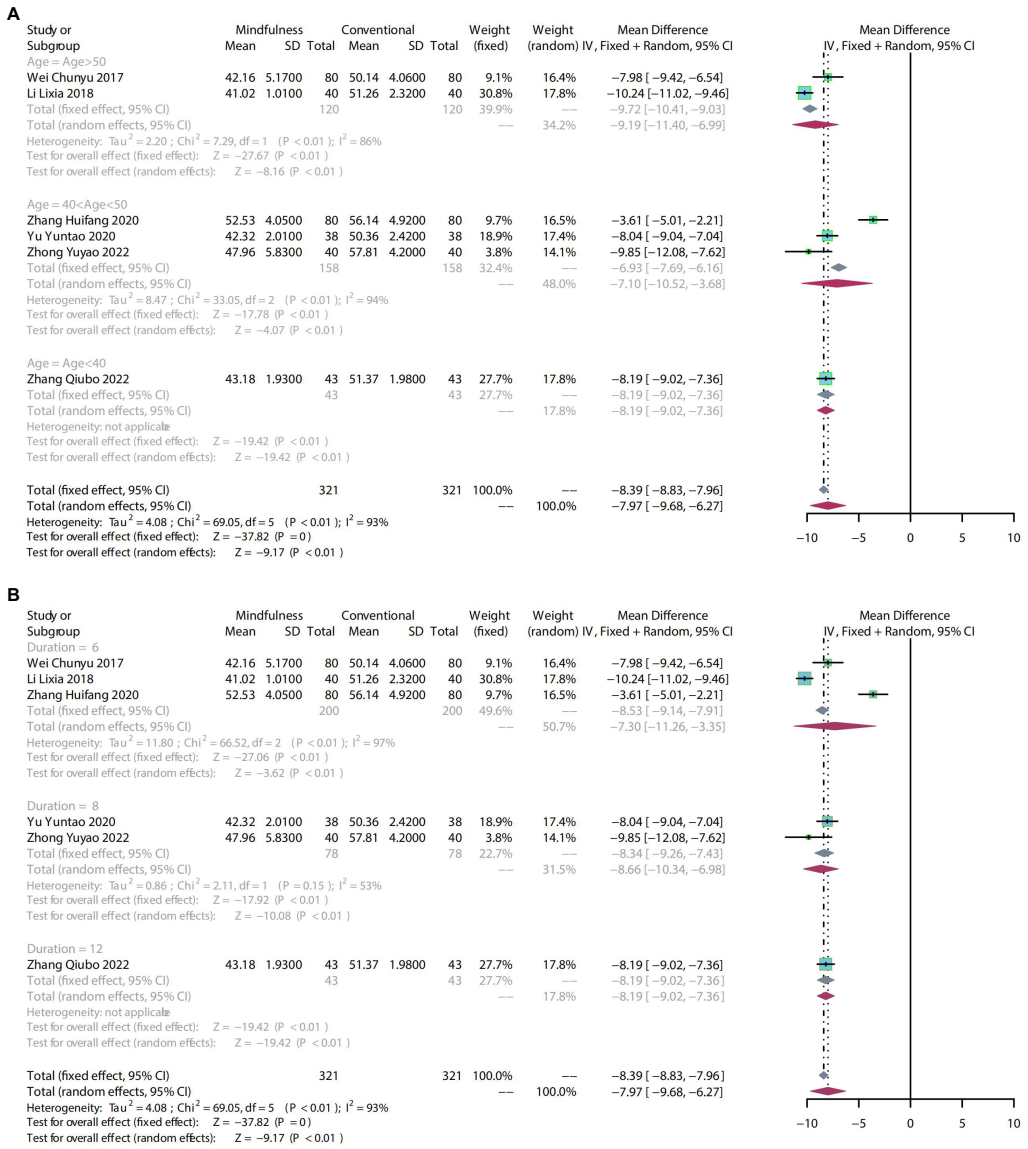
**(A)** forest plot shows the effect of mindfulness versus conventional treatment on SAS scores in subgroups stratified according to age. **(B)** The forest plot shows the effect of mindfulness versus conventional treatment on SAS scores in subgroups stratified according to duration of treatment.

To compare the effects of mindfulness therapy for anxiety in people with insomnia, anxiety and depression, six RCTs including 642 participants were analyzed to assess the endpoint SAS score. Due to the high heterogeneity (*p* < 0.01, *I^2^* = 93%), a random-effects model was used. The results showed a clear difference in reducing SAS scores between mindfulness treatment and conventional treatment (MD: −7.97, 95% CI: −9.68–−6.27, *p* < 0.01; Figure 7).

##### 3.4.4.2. Subgroup analysis

The SAS scores showed high heterogeneity (*p* < 0.01, *I^2^* = 93%).

After subgroup analysis by age, age > 50 (MD: −9.19, 95% CI: −11.40–−6.99, *p* < 0.01) and 40 < age > 50 (MD: −7.10, 95% CI: −10.52–−3.68, *p* < 0.01) demonstrated a significant difference compared with conventional treatment. Mindfulness therapy with age < 40 (MD: −8.19, 95% CI: −9.02–−7.36, *p* < 0.01) on impact of SAS scores may need more evidence to support it (Figure 7A).

The subgroups were divided into 6 weeks, 8 weeks, and 12 weeks according to the duration of treatment. 6 weeks (MD: −7.30, 95% CI: −11.26–−3.35, *p* < 0.01) and 8 weeks of mindfulness therapy (MD: −8.66, 95% CI: −10.34–−6.98, *p* < 0.01) had a significant difference compared to conventional treatment. The 12-week mindfulness treatment (MD: -8.19, 95% CI: −9.02–−7.36, *p* < 0.01) on the impact of SAS scores may need more evidence to support it (Figure 7B).

## 4. Discussion

### 4.1. Summary of findings

We conducted a systematic review and meta-analysis of 10 RCT trials including 1,058 participants. First, mindfulness therapy showed a higher improvement rate in improving anxiety and depression in people with insomnia, anxiety and depression compared with conventional treatment.

Secondly, for the primary outcomes HAMD and HAMA, analysis at both age and intervention time levels showed that mindfulness therapy had a positive correlation with the improvement of anxiety with age and intervention time. However, there is still relatively little clinical evidence and further research is needed. For the secondary outcomes SDS and SAS, mindfulness showed a positive effect on anxiety and depressive symptom self-ratings in insomnia patients with age and intervention time increased in subgroup analyses.

Finally, based on the primary and secondary outcomes, it can be concluded that the use of self-rating scales is more effective than physician ratings in reducing anxiety and depressive symptoms, but the level of specific clinical trial evidence needs to be further improved.

### 4.2. Mechanisms of mindfulness

Mindfulness belongs to the main method of cultivating the mind in Eastern Buddhist meditation, and is a concept of pursuing an ideal state and achieving the best balance between body and mind. Mindfulness training has a good effect on the treatment of various neuroses, addictive behaviors, personality disorders, etc., as well as in the general area of physical and mental health.

As for sleep, Kabat-Zinn (30) stated that mindfulness may help change sleep dysfunction, increase awareness, and correct poor sleep hygiene habits. Psychological distress is one of the important factors for persistent insomnia (31). The US DSM-V lists the clinical manifestations of depression as depressed mood, lack of interest or pleasure, decreased energy, or easy fatigue. Depression not only causes distress to the daily life of patients and their families, but also increases the probability of self-injury, suicide and cardiovascular disease, endangering the health and life safety of patients (32, 33). Anxiety is an unpleasant emotional experience that is extremely stressful and difficult to cope with. People may feel that something unfavorable seems to be about to happen. Anxiety symptoms fluctuate and may persist, increase and eventually affect social functioning and daily life (34). Many patients with anxiety disorders are also depressed, and those with severe symptoms are also at risk of suicide. Mindfulness-based interventions such as meditation, yoga, tai chi, and fitness qigong have been shown to be effective in relieving symptoms of depression and anxiety, improving emotional regulation, and regulating peripheral autonomic activity (blood pressure, heart rate, HRV) (35–44). This may be related to changes in higher cortical function by mindfulness interventions. Studies have shown that mindfulness and meditation can change brain structure, regulate physiological activities, and have positive significance for relieving psychological stress and autonomic nervous system balance (35, 45–48). The mechanism may be to regulate the output of sympathetic-parasympathetic tension by improving central activity under stress.

Mindfulness can affect brain structure changes, which may be the mechanism by which mindfulness can improve insomnia, anxiety and depression symptoms, but there are few relevant studies at present, which may be a hot spot for future psychiatric research.

### 4.3. Strengths and limitations

In our systematic review, instead of setting all adult people with insomnia as the research object, we analyzed the use of mindfulness therapy for people with insomnia accompanied by anxiety and depression symptoms. We found that the combination of mindfulness and conventional drug therapy was superior to conventional drug therapy alone in alleviating the severity of anxiety and depression symptoms. The results of this study involve all aspects of mindfulness and conventional drug combination therapy, such as the duration of mindfulness and the degree of adaptation in different age groups. This provides a more detailed treatment plan for mindfulness as an adjunct to the treatment of anxiety and depression symptoms.

There are still several limitations of this study: (1) The methodological quality of randomized controlled trials included in this study is low, and some articles do not specify blinding and specific interventions, which may lead to errors in the results of meta-analysis; (2) Few studies were conducted in this area, with only 10 RCTs included, which may have led to publication bias and low-certainty evidence for the results; (3) All trials were conducted in China, so it may be difficult to generalize the results to other countries and lead to publication bias; (4) In this meta-analysis, the observed benefit correlated only with the outcome at the end of treatment. The long-term effect of mindfulness alone or in combination with conventional drugs in the treatment of insomnia with anxiety and depression is unclear and deserves further study.

### 4.4. Implications

This systematic review and meta-analysis may have some potential implications for clinical practice. First of all, in the population of insomnia with anxiety and depression symptoms, the combination of mindfulness and conventional drug therapy is superior to conventional drug therapy alone in alleviating the severity of anxiety and depression symptoms, and has important practical clinical value in reducing the number of patients using anti-anxiety-depressive drugs. Therefore, based on the evidence of this study, we recommend that patients with insomnia who are receiving standard anti-anxiety-depressive drugs, especially those who respond poorly to anti-anxiety-depressive drugs and cannot tolerate it, can also receive mindfulness treatment. When receiving mindfulness therapy, the remission of anxiety and depression symptoms in older patients with longer treatment duration will be more obvious. Because the randomized controlled trials included in this study showed that there were differences in the mindfulness operation methods, treatment frequency, treatment duration and the degree of adaptation of people of different ages in different trials, the best mindfulness treatment prescription for insomnia with anxiety and depression is still unclear.

Clinical trials on anxiety and depression symptoms should employ uniform diagnosis, outcome selection and reporting criteria, which will be helpful for comparisons among studies; furthermore, to mitigate the psychological component of subjective metrics, objective metrics related to anxiety and depression, such as circulating 5-HT (49), cortisol and adrenocorticotropic hormone (50) levels, and electroencephalography (51), could be used to evaluate the anti-anxiety-depressive effects of mindfulness. Future work should combine high-quality and large-scale randomized controlled trials with meta-analysis to study the efficacy and safety of mindfulness in the treatment of anxiety and depression symptoms in insomnia patients, and to determine the best treatment.

## 5. Conclusion

Based on the results of this systematic review and meta-analysis, people of different ages with insomnia, anxiety and depression can add 4–12 weeks of mindfulness therapy on the basis of conventional treatment, which has a significant effect on the improvement of anxiety and depressive symptoms. This is a new diagnosis and treatment idea recommended for insomnia patients with or without anxiety and depression symptoms. Due to methodological flaws in the included studies and the limited sample size of this article, the results of our meta-analysis should be considered with caution. In the future clinical research, we should pay attention to the standardization and scientific design of randomized controlled trials, and carry out multi-center, large sample and sufficient follow-up time, so as to obtain more systematic, objective and accurate curative effect, and provide reliable evidence for further proving the superiority of mindfulness in the treatment of neuropsychiatric symptoms such as anxiety and depression in insomniacs.

## Data availability statement

The original contributions presented in the study are included in the article/Supplementary material, further inquiries can be directed to the corresponding author/s.

## Ethics statement

The study does not involve patient privacy or rights and does not require ethics committee approval. Results can be published in peer-reviewed journals or disseminated at relevant conferences.

## Author contributions

HL: design, conception, performing statistical analysis, interpreting data, drafting the manuscript. WQ: conception, revising manuscript, searching the literature, extracting data, evaluating quality. NL and WS: conception, performing statistical analysis, drafting the manuscript. SF: searching the literature, extracting data, evaluating quality. JW: searching the literature, evaluating quality. YZ, TW, CW, and XC: extracting data, evaluating quality. YS: revising manuscript. DH: design, conception, revising the manuscript, interpreting data. YL: design, conception, revising manuscript. All authors contributed to the article and approved the submitted version.

## Funding

This work was supported by the National Key R&D Program of China (No. 2019YFC1709801).

## Conflict of interest

The authors declare that the research was conducted in the absence of any commercial or financial relationships that could be construed as a potential conflict of interest.

## Publisher’s note

All claims expressed in this article are solely those of the authors and do not necessarily represent those of their affiliated organizations, or those of the publisher, the editors and the reviewers. Any product that may be evaluated in this article, or claim that may be made by its manufacturer, is not guaranteed or endorsed by the publisher.
