# Supplementary material for: Effect of mindfulness on anxiety and depression in insomnia patients: A systematic review and meta-analysis
Source: Front Psychiatry. 2023 Mar 2;14:1124344. doi: 10.3389/fpsyt.2023.1124344 (PMC10018191; doi:10.3389/fpsyt.2023.1124344)
Supplement: Supplementary file 1 [file Data_Sheet_1.docx]

**Search Strategies**

The search was conducted using a combination of subject terms and free terms, and was adjusted to the characteristics of each database. References included in the study were also searched to supplement access to relevant information. Search terms include: insomnia、sleep disorder、anxiety、depression、negative emotion、 Mindfulness、Randomized Controlled Trial etc. Take PubMed as an example, its specific search strategy is:

| #1 insomnia[MeSH Terms] |
| --- |
| #2 insomnia[Title/Abstract] |
| #3 sleep disorder[MeSH Terms] |
| #4 sleep disorder[Title/Abstract] |
| #5 sleep disorders[MeSH Terms] |
| #6 sleep disorders[Title/Abstract] |
| #7 sleep disturbance[MeSH Terms] |
| #8 sleep disturbance[Title/Abstract] |
| #9 sleep problem[MeSH Terms] |
| #10 sleep problem[Title/Abstract] |
| #11 sleep problems[MeSH Terms] |
| #12 sleep problems[Title/Abstract] |
| #13 Sleeplessness[MeSH Terms] |
| #14 Sleeplessness[Title/Abstract] |
| #15 agrypnia[MeSH Terms] |
| #16 agrypnia[Title/Abstract] |
| #17 #1 OR #2 OR #3 OR #4 OR #5 OR #6 OR #7 OR #8 OR #9 OR #10 OR #11 OR #12 OR #13 OR #14 OR #15 OR #16 |
| #18 anxiety[MeSH Terms] |
| #19 anxiety[Title/Abstract] |
| #20 anxious[MeSH Terms] |
| #21 anxious[Title/Abstract] |
| #22 anxiety state[MeSH Terms] |
| #23 anxiety state[Title/Abstract] |
| #24 anxiety neurosis[MeSH Terms] |
| #25 anxiety neurosis[Title/Abstract] |
| #26 anxiety disorder[MeSH Terms] |
| #27 anxiety disorder[Title/Abstract] |
| #28 anxiety disorders[MeSH Terms] |
| #29 anxiety disorders[Title/Abstract] |
| #30 anxiety symptom[MeSH Terms] |
| #31 anxiety symptom[Title/Abstract] |
| #32 depression[MeSH Terms] |
| #33 depression[Title/Abstract] |
| #34 depressive state[MeSH Terms] |
| #35 depressive state[Title/Abstract] |
| #36 depression state[MeSH Terms] |
| #37 depression state[Title/Abstract] |
| #38 depressive[MeSH Terms] |
| #39 depressive[Title/Abstract] |
| #40 depressed[MeSH Terms] |
| #41 depressed[Title/Abstract] |
| #42 depressive symptoms[MeSH Terms] |
| #43 depressive symptoms[Title/Abstract] |
| #44 depression disorder[MeSH Terms] |
| #45 depression disorder[Title/Abstract] |
| #46 negative emotion[MeSH Terms] |
| #47 negative emotion[Title/Abstract] |
| #48 negative emotions[MeSH Terms] |
| #49 negative emotions[Title/Abstract] |
| #50 emotional disorder[MeSH Terms] |
| #51 emotional disorder[Title/Abstract] |
| #52 mood disorders[MeSH Terms] |
| #53 mood disorders[Title/Abstract] |
| #54 #18 OR #19 OR #20 OR #21 OR #22 OR #23 OR #24 OR #25 OR #26 OR #27 OR #28 OR #29 OR #30 OR #31 OR #31 OR #32 OR #33 OR #34 OR #35 OR #36 OR #37 OR #38 OR #39 OR #40 OR #41 OR #42 OR #43 OR #44 OR #45 OR #46 OR #47 OR #48 OR #49 OR #50 OR #51 OR #52 OR #53 |
| #55 Mindfulness-based stress reduction[MeSH Terms] |
| #56 Mindfulness-based stress reduction[Title/Abstract] |
| #57 MBSR[MeSH Terms])) OR (MBSR[Title/Abstract] |
| #58 mindfulness-based intervention[MeSH Terms] |
| #59 mindfulness-based intervention[Title/Abstract] |
| #60 Meditative movement[MeSH Terms] |
| #61 Meditative movement[Title/Abstract] |
| #62 Mindfulness[MeSH Terms] |
| #63 Mindfulness[Title/Abstract] |
| #64 mindful[MeSH Terms] |
| #65 mindful[Title/Abstract] |
| #66 Meditation[MeSH Terms] |
| #67 Meditation[Title/Abstract] |
| #68 meditational exercise[MeSH Terms] |
| #69 meditational exercise[Title/Abstract] |
| #70 sitting quietly[MeSH Terms] |
| #71 sitting quietly[Title/Abstract] |
| #72 sitting silently[MeSH Terms] |
| #73 sitting silently[Title/Abstract] |
| #74 sitting crosslegged[MeSH Terms] |
| #75 sitting crosslegged[Title/Abstract] |
| #76 Yoga[MeSH Terms] |
| #77 Yoga[Title/Abstract] |
| #78 stake standing[MeSH Terms] |
| #79 stake standing[Title/Abstract] |
| #80 Taiji[MeSH Terms] |
| #81 Taiji[Title/Abstract] |
| #82 Taijiquan[MeSH Terms] |
| #83 Taijiquan[Title/Abstract] |
| #84 Tai chi[MeSH Terms] |
| #85 Tai chi[Title/Abstract] |
| #86 Qigong[MeSH Terms] |
| #87 Qigong[Title/Abstract] |
| #88 mind-body therapy[MeSH Terms] |
| #89 mind-body therapy[Transliterated Title] |
| #90 #55 OR #56 OR #57 OR #58 OR #59 OR #60 OR #61 OR #61 OR #62 OR #63 OR #64 OR #65 OR #66 OR #67 OR #68 OR #69 OR #70 OR #71 OR #72 OR #73 OR #74 OR #75 OR #76 OR #77 OR #78 OR #79 OR #80 OR #81 OR #82 OR #83 OR #84 OR #85 OR #86 OR #87 OR #88 OR #89 |
| #91 randomized controlled trial[Publication Type] |
| #92 randomized[Title/Abstract] |
| #93 placebo[Title/Abstract] |
| #94 Controlled Clinical Trial[Publication Type] |
| #95 #91 OR #92 OR #93 OR #94 |
| #96 #17 AND #54 AND #90 AND #95 |
